# Mental health of Japanese psychiatrists: the relationship among level of occupational stress, satisfaction and depressive symptoms

**DOI:** 10.1186/s13104-015-1054-7

**Published:** 2015-03-26

**Authors:** Akihiro Koreki, Atsuo Nakagawa, Akiko Abe, Hidetsugu Ikeuchi, Jo Okubo, Atsushi Oguri, Keisuke Orimo, Nariko Katayama, Hiroyo Sato, Ryo Shikimoto, Go Nishiyama, Waka Nogami, Kazuma Haki, Tetsuro Hayashi, Yuko Fukagawa, Kei Funaki, Mia Matsuzawa, Ayako Matsumoto, Masaru Mimura, for the Keio Psychiatry Resident Class of 2008

**Affiliations:** Department of Neuropsychiatry, Keio University School of Medicine, Tokyo, Japan; Center for Clinical Research, Keio University School of Medicine, Tokyo, Japan

**Keywords:** Occupational satisfaction, Stress, Workplace, Depression, Resilience

## Abstract

**Background:**

Psychiatrists in clinical practice face a number of stressors related to patient care, such as overwork. On the other hand, they gain satisfaction from their work. We quantified and assessed the potential relationship between levels of occupational stress, satisfaction, and depressive symptoms among Japanese clinical psychiatrists. We surveyed 206 psychiatrists with up to 15 years of clinical experience who primarily worked in patient care. Levels of occupational stress and occupational satisfaction were measured using the Visual Analogue Scale and the level of depressive symptoms was measured by the Center for Epidemiologic Studies Depression Scale. Workplace stressors and satisfiers were also evaluated.

**Results:**

Out of 206 psychiatrists, 154 (74.8%) responded to the survey. The respondents’ mean (SD) age was 34.3 (5.2) years. The estimated prevalence of significant depressive symptoms was 34.4% (n = 53), and the experienced frequent violence was 14.9% (n = 23). The level of depressive symptoms was inversely correlated with the level of occupational satisfaction. In respondents who reported a moderate level of occupational stress, having fewer depressive symptoms was associated with higher occupational satisfaction, but this association was not significant in those who reported a high level of stress. In addition, high occupational satisfaction was associated with interest towards work content, ability to work at one’s discretion, opportunities for growth and career development, and ease of communication with supervisors and colleagues.

**Conclusions:**

Nearly one-third of the psychiatrists screened positive for significant depressive symptoms. Having fewer depressive symptoms was associated with higher occupational satisfaction in those who reported a moderate level of stress. Implications from the present findings may be to enhance occupational satisfaction by discussing work interests with a supervisor, as well as increased opportunities for career development, which may prevent depression among psychiatrists.

**Electronic supplementary material:**

The online version of this article (doi:10.1186/s13104-015-1054-7) contains supplementary material, which is available to authorized users.

## Background

Healthcare workers such as physicians and nurses have been associated with greater occupational risk of developing psychiatric morbidity, specifically depression [1]. This is partly because healthcare workers in general are exposed to many occupational stressors, such as work overload, receiving a limited amount of support, and poor management [2]. Healthcare workers under chronic occupational stress are at a high risk for developing depression, which is mediated through burnout [2-7]. Further, a large survey of U.S. anesthesiology residents revealed that residents who exhibited a high risk of depression reported a 30-fold higher frequency of committing multiple medical errors compared to those with a lower risk of depression [8]. Previous studies have reported that depressed physicians’ performance is associated with suboptimal care and more medical errors [2,9,10]. Thus, physicians’ stress-related depressive symptoms not only affects individuals’ health, but also has a great adverse impact on their professional skills, including patient care.

Occupational satisfaction is also an important factor for preventing burnout, which has been found to be a precursor of depressive symptoms. Although burnout and depression share similar dysphoric symptoms due to conceptual overlap [11,12], each is thought to have a distinct construct. Maslach [13] described burnout as a syndrome of emotional exhaustion, depersonalization, and reduced personal accomplishment, which is confined to the work environment. In contrast, clusters of severe and pervasive symptoms that affect multiple domains of an individual’s life characterize depression [12]. Previous studies [14,15] have shown that physician burnout is positively associated with levels of occupational stress and inversely associated with occupational satisfaction. Interestingly, Ramirez et al. [15] demonstrated that, if under similar levels of occupational stress, physicians with higher levels of occupational satisfaction were protected from burnout. Moreover, occupational satisfaction strongly correlates with occupational performance [16]. A previous study demonstrated the association between occupational satisfaction and a reduction in medical error reports in hospitals [17]. Thus, higher levels of occupational satisfaction may function as a buffer against high turnover among healthcare workers [18]. Taken together, these findings may imply that occupational satisfaction can be an important factor for preventing the development of depressive symptoms.

**Figure 1:**
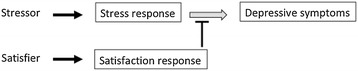
**Theoretical model of stress-depression process.** Exposure to stressors may produce stress response, and excessive amount of stress response may develop depressive symptoms. On the other hand, satisfier may produce satisfaction response. Satisfactory response may have protective effect against the progression of depressive symptoms.

Psychiatrists have been consistently shown to be at a higher risk for depression and suicide compared with other healthcare workers [19]. Psychiatrists in clinical practice face a number of stressors related to patient care such as overwork, inadequate resources, and poor interactions with staff and work colleagues [20]. Further, the majority of psychiatrists experience threatening situations, such as violent patients and incidences of patient suicide during the course of their careers [21-23]. Despite the importance of depressive symptoms, occupational stress, and occupational satisfaction among healthcare workers, little is known about the potential relationships among these factors in psychiatrists. We therefore aimed to quantify the level of depressive symptoms, occupational stress, and occupational satisfaction and assessed the potential relationship of these factors among Japanese psychiatrists. Although some previous studies focus on burnout [15,24], we focused on depressive symptoms, because physicians’ depression is known to be quite prevalent and to have an adverse impact on patient care [2,9,19,25]. Hypothetical model (Figure 1) was proposed with reference to theoretical framework [6] and above empirical research findings. The purpose of the present study was to clarify this relationship between the level of depressive symptoms, occupational stress, and occupational satisfaction. We also examined additional potential factors that may influence occupational stress and satisfaction.

## Methods

### Participants and data collection

In February 2010, an anonymous self-administered survey was sent to participants using a convenience sample of psychiatrists working at a university hospital located in central Tokyo, as well as 15 affiliated teaching hospitals: 5 general hospitals, and 10 psychiatric hospitals. Of the 15 study sites, seven were located in suburban Tokyo, and eight were in the Greater Tokyo area. Because we were predominantly interested in the link between occupational stress and patient care rather than administrative issues, psychiatrists who had up to 15 years of clinical experience and who primarily worked in patient care were eligible to participate in the present study.

### Survey measures

#### Demographic and workplace characteristics

We collected demographic and workplace characteristics from the participants, including age, gender, post-graduate year (PGY), licensure status of designated psychiatrists (referred to as *seishin hoken shitei-i*, a psychiatrist authorized under the Mental Health and Welfare Law), current work setting, number of working hours per day, number of outpatients seen per day, and the daily number of inpatients in their care.

#### Level of depressive symptoms

The Japanese version of the Center for Epidemiologic Studies Depression Scale (CES-D) was used to evaluate the level of depressive symptoms [26,27]. CES-D is a 20-item questionnaire with each item scored using a four-point rating scale ranging from 0–3. The CES-D (total) scores range from 0–60, with higher scores indicating a higher severity of depressive symptoms. The standard cut-off score of ≥16 yielded a sensitivity of 0.95 and specificity of 0.70 in predicting Major Depressive Disorder (MDD) [28].

#### Level of occupational stress

Selye described “stress” as a “stress response”, which is thought to be a different construct from a “stressor” [29]. Stress response is a physical condition involving high cortisol and sympathetic nervous system activation, while a stressor is a factor that leads to a stress response. In the present study, we defined occupational stress as a stress response. Although previous studies evaluated stress by the total number of stressors [30,31], in the present study, a global level of occupational stress (i.e., stress response) was quantified by using the Visual Analogue Scale (VAS). Several studies have used VAS to quantify the level of occupational stress [15,24,32]. We asked participants to indicate their stress levels on a scale from 0–100, with higher scores indicating higher levels of stress.

#### Level of occupational satisfaction

Similar to the level of occupational stress, overall level of occupational satisfaction was quantified with VAS. We asked participants to indicate their level of occupational satisfaction using a scale from 0–100, with higher scores indicating greater satisfaction.

#### Workplace stressors and satisfiers

We evaluated workplace stressors and satisfiers for all participating psychiatrists. Based on Herzberg’s motivation-hygiene theory [33], dissatisfiers (i.e., stressors) and motivators (i.e., satisfiers) partly overlap, but are distinct constructs. We evaluated workplace stressors by using a four-point Likert scale derived from the job stressor subscale taken from 12-item abbreviated version of the Brief Job Stress Questionnaire (BJSQ) [31], instead of using the total score. The items assessed are as follows: (1) Work overload, (2) insufficient time, (3) necessity to concentrate one’s attention on work (job demand factor), (4) ability to work at one’s discretion, (5) ability to make one’s own decisions, (6) ability to reflect on one’s opinion (job control factor), (7) ease of communication with a supervisor, (8) ability to rely on a supervisor, (9) supervisor spends time on one’s personal issues (supervisor support factor), (10) ease of communication with colleagues, (11) ability to rely on colleagues, and (12) colleagues spending time on each other’s personal issues (collegial support factor). Additionally, we included workplace security as an additional stressor because the majority of psychiatrists experience threatening situations, such as managing agitated and violent patients within their workplace [34-36] (see Additional file 1).

We also evaluated workplace satisfiers using a five-point Likert scale derived from the occupational satisfaction motivating factors identified in Van Saane’s systematic review [37]. Initially, all eleven occupational satisfiers identified in Van Saane’s systematic review were considered for workplace satisfiers, including autonomy, work contents, communication, monetary rewards, opportunity for growth and career development, promotion, co-workers, meaningfulness (gratitude and respect from patients), supervision, workload, and work demands; however, we excluded six of the factors that were a direct opposite of a workplace stressor. For example, we decided “promotion” would be irrelevant for assessing a sample of clinical psychiatrists, thus we excluded this factor. As a result, the following four factors were determined as workplace satisfiers for use in the present study: (1) work content (i.e., interest and accomplishment towards work), (2) monetary rewards (i.e., salary), (3) opportunity for growth and career development (i.e., participating productively in conferences and lectures), and (4) meaningfulness (i.e., gratitude and respect from patients) [38].

### Statistical analysis

Pearson and Spearman correlation coefficients were used to determine the association between variables. To clarify the relationship between reported levels of depressive symptoms and the level of occupational satisfaction under various stress levels, we stratified occupational stress into three levels (by the tertile of Stress VAS score), and compared level of depressive symptoms (CES-D score) with groups of high, moderate, and low occupational satisfaction (by the tertile of Satisfaction VAS score) using analysis of variance (ANOVA) with a Bonferroni correction.

Next, to clarify the potential factors that could influence occupational stress and satisfaction, each factor was evaluated using univariate analysis. Workplace stressors and workplace satisfiers were categorized into binary outcomes (higher versus lower). The higher stressor subgroup was defined by combining the “agree” or “somewhat agree” responses in the questionnaire and the higher satisfier subgroup was defined by combining the “always” or “often” responses in the questionnaire. Student’s t-tests were conducted to compare the Stress or the Satisfaction VAS scores between the higher and lower subgroups, respectively. Subsequently, the strength of the relationships between the dependent and independent variables were examined using stepwise multiple regression analyses. Dependent variables were the Stress VAS score and the Satisfaction VAS score, respectively. Independent variables were the variables that demonstrated a significant association with the Stress VAS score or the Satisfaction VAS score in the univariate analyses. Significance for two-tailed tests was set at p < 0.05. Statistical analyses were carried out using SPSS Version 21.0 (IBM Corp., Armonk, NY, USA).

### Ethical considerations

Ethical approval was obtained from the Institutional Ethical Review Board of the Keio University School of Medicine.

## Results

We surveyed 206 psychiatrists and 74.8% responded (n = 154). The majority of respondents were male (n = 111, 72.1%) and the mean (SD) age of participants was 34.3 (5.2) years. Of the total respondents, the mean (SD) period of post-graduate (PGY) training was 5.5 (4.0) years. Additionally, 41.6% (n = 64) of the sample were licensed psychiatrists. More than half of the respondents were working at psychiatric hospitals (n = 92, 59.7%), followed by university hospitals (n = 35, 22.7%), general hospitals (n = 21, 13.6%), and community psychiatric clinics (n = 6, 3.9%). The mean (SD) working hours per day were 8.3 (1.9) hours and the mean (SD) number of outpatients seen per day was 26.0 (10.7). For respondents who have been working at a hospital, the mean (SD) number of inpatients in their care was 15.7 (12.8). Approximately 14.9% (n = 23) of the psychiatrists had experienced frequent violence during patient care. There was no significant association between the level of depressive symptoms with gender, age, or the type of hospital, the number of outpatients seen per day. Further, there was no significant association between the levels of occupational stress and satisfaction (measured by VAS) with these factors (data available upon request).

### Level of depressive symptoms, level of occupational stress, and level of occupational satisfaction

Among the respondents, 34.4% (n = 53) had significant depressive symptoms (CES-D score ≥16) and the mean (SD) CES-D score was 13.4 (9.2). The mean (SD) levels of occupational stress (Stress VAS score) and occupational satisfaction (Satisfaction VAS score) were 53 (22.8) and 68.8 (14.2), respectively. Level of depressive symptoms (CES-D score) was positively correlated with the level of occupational stress (Stress VAS score) (r = 0.52, p < 0.001) and inversely correlated with the level of occupational satisfaction (Satisfaction VAS score) (r = −0.36, p < 0.001). Level of occupational stress (Stress VAS score) showed an inverse correlation with level of occupational satisfaction (Satisfaction VAS score) (r = −0.33, p < 0.001).

### Comparison of level of depressive symptoms among high, moderate, and low level of occupational satisfaction and stress

**Figure 2:**
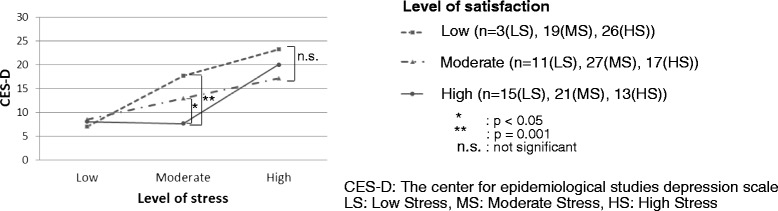
**The relationship between level of depressive symptoms and level of occupational satisfaction under the three levels of occupational stress.** Occupational stress was stratified by the tertile of Stress VAS score. Level of depressive symptoms was measured with the CES-D. Level of satisfaction was categorized into three groups (high, moderate, and low) by the tertile of Satisfaction VAS score. In respondents with a moderate level of stress, less severe depressive state was associated with both high and moderate occupational satisfaction (p = 0.001, p < 0.05, respectively).

The comparison of level of depressive symptoms (CES-D score) among high, moderate, and low occupational satisfaction groups illustrated by the three levels of occupational stress are presented in Figure 2. In respondents who reported a moderate level of stress, less severe depressive state was associated with both high and moderate occupational satisfaction (p = 0.001, 0.047, respectively). Interestingly, this association was not found among respondents with high or low levels of occupational stress (high occupational stress: F (2,46) = 1.724, p = 0.19; low occupational stress: F (2,52) = 7.358, p = 0.92).

### Factors associated with level of occupational stress and satisfaction

Table 1 presents a comparison between high versus low levels of occupational stress and satisfaction with workplace stressors and satisfiers. High levels of occupational stress were associated with work overload, insufficient time, inability to work at one’s discretion, inability to make one’s own decisions, inability to reflect on one’s opinion, inability to rely on colleagues, and workplace insecurity. In contrast, high occupational satisfaction was associated with interest towards work content, opportunities for growth and career development, gratitude and respect from patients, ability to work at one’s discretion, ability to make one’s own decisions, ability to reflect on one’s opinion, ease of communication with supervisors, ability to rely on supervisors, ease of communication with colleagues, and colleagues spend time on one’s personal issues.

**Table 1:** **Univariate associations with level of stress and satisfaction by high and low stressors and satisfiers**

	**Level of occupational stress p**			**Level of occupational satisfaction p**		
	**(Stress VAS score)**			**(Satisfaction VAS score)**		
	**Mean (SD)**	**N**	**p** ^**a**^	**Mean (SD)**	**N**	**p** ^**a**^
**Stressors (general)** ^**b**^						
Job demand factor:						
Work overload						
High	60.1 (21.5)	84	<0.01	68.4 (14.2)	84	0.73
Low	44.4 (21.0)	68		69.2 (14.3)	68	
Insufficient time						
High	48.9 (22.6)	96	0.03	70.9 (13.8)	96	0.12
Low	60.2 (21.1)	56		65.0 (14.2)	56	
Necessity to concentrate one’s attention on work						
High	52.7 (22.7)	130	0.94	69.2 (14.1)	130	0.40
Low	53.0 (25.3)	23		66.5 (14.7)	23	
Job control factor:						
Ability to work at one’s discretion						
High	47.1 (21.3)	106	<0.01	72.3 (12.9)	106	<0.01
Low	66.7 (19.4)	46		60.7 (13.9)	46	
Ability to make one’s own decisions						
High	50.0 (23.0)	122	<0.01	71.6 (12.9)	122	<0.01
Low	63.2 (19.7)	31		57.7 (13.5)	31	
Ability to reflect on one’s opinions						
High	48.0 (21.9)	109	<0.01	71.2 (12.9)	109	<0.01
Low	64.3 (21.7)	44		63.1 (15.6)	44	
Supervisor support factor:						
Ease of communication with a supervisor						
High	51.1 (24.0)	98	0.16	71.9 (14.0)	98	<0.01
Low	56.5 (20.1)	54		63.2 (13.0)	54	
Ability to rely on a supervisor						
High	51.5 (22.0)	106	0.20	70.7 (64.2)	106	0.011
Low	56.7 (24.5)	45		64.2 (13.9)	45	
Supervisor spends time on one’s personal issues						
High	51.1 (22.6)	100	0.13	70.1 (14.6)	100	0.10
Low	56.9 (23.0)	51		66.1 (13.3)	51	
Collegial support factor:						
Ease of communication with colleagues						
High	50.4 (23.8)	108	0.16	71.6 (13.7)	108	<0.01
Low	59.3 (18.6)	44		62.1 (13.2)	44	
Ability to rely on colleagues						
High	49.3 (23.4)	93	0.012	70.3 (14.8)	93	0.10
Low	58.8 (20.6)	59		66.4 (13.0)	59	
Colleagues spend time on one’s personal issues						
High	50.6 (23.9)	94	0.10	71.2 (14.6)	94	<0.01
Low	56.9 (20.4)	58		65.0 (12.9)	58	
**Stressors (specific)**						
Workplace insecurity:						
High	50.4 (21.6)	23	<0.01	70.4 (15.5)	23	0.56
Low	67.6 (24.0)	129		68.5 (14.0)	129	
**Satisfiers**						
Interest towards work contents:						
High	51.6 (3.9)	70	0.48	74.5 (11.8)	70	<0.01
Low	54.2 (21.8)	82		64.0 (14.4)	82	
Opportunity for growth and career development:						
High	50.3 (22.8)	75	0.15	72.1 (13.1)	75	<0.01
Low	55.9 (23.2)	68		65.9 (14.8)	68	
Monetary reward:						
High	52.7 (23.2)	64	0.92	69.8 (14.4)	64	0.53
Low	53.1 (23.0)	86		68.4 (13.9)	86	
Gratitude and respect from patients:						
High	47.8 (23.4)	23	0.27	74.8 (15.3)	23	0.03
Low	53.6 (22.9)	129		67.9 (13.7)	129	

BJSQ: Brief Occupational Stress Questionnaire, VAS: Visual Analogue Scale.

^a^t-test.

^b^Derived from the 12-item abbreviated version BJSQ job stressor subscale.

### Multiple regression analyses

We conducted stepwise multiple regression analysis using level of occupational stress (Stress VAS score) as the dependent variable. Significant factors in the bivariate tests included the following independent variables: work overload, insufficient time, inability to work at one’s discretion, inability to make one’s own decisions, inability to reflect on one’s opinion, inability to rely on a colleague, and workplace insecurity. Stepwise multiple regression analysis showed that level of occupational stress was significantly associated with an overload of work (p = 0.001), inability to work at one’s discretion (p = 0.001), inability to reflect on one’s opinions (p = 0.009), and inability to rely on colleagues (p = 0.014) (see Table 2). This regression model explained 27.8% of the variance found in the level of stress variable (adjusted R^2^ = 0.28, F = 15.35, df = 4.145, p < 0.001).

**Table 2:** **Stressors associated with level of occupational stress (Stress VAS score): results of stepwise multiple regression analysis (final model)**

	**beta**	**t**	**p**
Stressors (general)			
Job demand factor			
Work overload	0.24	3.38	0.001
Job control factor			
Ability to work at one’s discretion	−0.26	−3.52	0.001
Ability to reflect on one’s opinions	−0.20	−2.65	0.009
Collegial support factor			
Ability to rely on colleagues	−0.18	−2.48	0.014
Total R^2^	0.30		
Adjusted R^2^	0.28		

VAS: Visual Analogue Scale.

Subsequently, we conducted stepwise multiple regression analyses with level of occupational satisfaction (Satisfaction VAS score) as the dependent variable. The independent variables were factors associated with significant effects in the bivariate analyses as follows: the ability to work at one’s discretion, ability to make one’s own decisions, ability to reflect on one’s opinion, ease of communication with supervisors, ability to rely on supervisors, ease of communication with colleagues, colleagues spend time on one’s personal issues, interest towards work content, opportunities for growth and career development, and gratitude and respect from patients. Stepwise multiple regression analysis showed that level of occupational satisfaction was significantly associated with having an interest towards work (p = 0.001), ability to work at one’s discretion (p < 0.001), opportunities for growth and career development (p = 0.03), ease of communication with supervisors (p = 0.04), and ease of communication with colleagues (p = 0.043) (see Table 3). This regression model revealed 33.8% of the variance in the level of satisfaction variable (adjusted R^2^ = 0.34, F = 15.28, df = 5.135, p < 0.001).

**Table 3:** **Satisfiers associated with level of occupational satisfaction (Satisfaction VAS score): Results of stepwise multiple regression analysis (final model)**

	**beta**	**t**	**p**
Stressors (general)			
Job control factor			
Ability to work at one’s discretion	0.31	4.24	<0.001
Supervisory support factor			
Ease of communication with a supervisor	0.17	2.08	0.04
Collegial support factor			
Ease of communication with colleagues	0.17	2.05	0.04
Satisfiers			
Interest towards work contents	0.25	3.36	0.001
Opportunity for growth and career development	0.15	2.16	0.03
Total R^2^	0.36		
Adjusted R^2^	0.34		

VAS: Visual Analogue Scale.

## Discussion

We conducted a survey to quantify and assess the potential relationship among depressive symptoms, occupational stress, and occupational satisfaction in Japanese clinical psychiatrists. The response rate for the present survey study was relatively high. We observed that one-third of the clinical psychiatrists surveyed had significant depression symptomatology and nearly 15% of psychiatrists surveyed had frequent experiences with violent patients during care. Although there are some differences in study designs and sample populations, the estimated prevalence of significant depressive symptomatology based on CES-D scores of 16 or higher among Japanese psychiatrists in the present study (33.4%) was slightly higher compared to the prevalence of 27% reported among British consultants [15] and 22% among American trainees in anesthesiology [8]. Indeed, a nationwide survey of Japanese psychiatrists identified more than 70% of respondents who experienced low levels of personal accomplishments, a key domain of burnout, and a precursor for depression [39].

We also found that lower levels of depressive symptoms were associated with higher occupational satisfaction in psychiatrists who reported a moderate level of occupational stress. This association was consistent with previous findings from UK and Dutch studies that examined physicians in other medical specialties [15,24].

In keeping with the concept of depression and satisfaction, level of occupational satisfaction showed an inverse correlation with the level of depressive symptoms in the present study. We also found that higher occupational satisfaction was associated with lower depression in psychiatrists who reported moderate levels of occupational stress. Interestingly, we did not find this association in those who reported a high level of occupational stress. This unique association between occupational satisfaction and depression may imply that occupational satisfaction may protect against the worsening of depressive symptoms among psychiatrists under moderate levels of occupational stress. From the standpoint of occupational hygiene, under extremely high-stress environments, we should first reduce the level of occupational stress to (at least) a moderate level. Then, we should focus on improving the level of occupational satisfaction to prevent depressive symptoms from emerging. Ramirez [15] asserted that jobs with high demands such as physicians, autonomy, and the feeling of being well-managed and resourced are important determinants of occupational satisfaction. The historical Herzberg [33] two-factor theory asserted that factors generating occupational dissatisfaction and satisfaction were distinct. Herzberg noted that “hygiene factors” (e.g., working condition and human relationships) produced dissatisfaction and “motivating factors” (e.g., achievement and growth) led to satisfaction. Additionally, Visser and colleagues [24] reported that intellectual stimulation among medical specialists was associated with occupational satisfaction, but not with stress. Hence, when developing a prevention program for depression among mental health workers, it may be unrealistic to largely alter “hygiene factors”, but we may focus more on “motivating factors” or resilience factors [40]. Notably, previous studies have suggested that positive emotions are considered to be a factor of resilience in depression [41].

In our study, high occupational satisfaction was associated with interest in work, opportunities for growth and career development, the ability to work at one’s own discretion, and the ability to communicate with supervisors and colleagues with ease. These findings imply that in order to improve occupational satisfaction among psychiatrists, administrators should make an effort to individually discuss work interests, such as clinical interests, and opportunities for career development with their staff. For example, if a certain psychiatrist is interested in ambulatory psychogeriatric care, providing an opportunity to work in outpatient geriatric mental health services may help to improve their occupational satisfaction. Previous studies have demonstrated that high occupational satisfaction due to a greater interest in work leads to higher clinical performance [16,17]. Opportunity for growth and career development is also an important determinant of occupational satisfaction. Although the present survey studied younger psychiatrists who were affiliated with university teaching hospitals, administrators should be cognizant of the great importance in providing continuing medical educational in order to develop young psychiatrists’ careers. Of note, we did not find a significant association between salary and occupational satisfaction. Therefore, to enhance occupational satisfaction, administrators should focus on psychiatrists’ intrinsic motivating factors, such as clinical interests and opportunities for career development, rather than monetary rewards.

The present study’s findings should be considered with a few limitations in mind. First, the sample was relatively small and selective, which limits the generalizability of the present findings. Our participants were mostly young physicians affiliated with university teaching hospitals who may have a high interest in career development. Second, the present study employed a cross-sectional design; thus, caution must be exercised in the interpretation of the observed associations. Although response bias is always a concern in cross-sectional studies, we obtained a relatively high response rate. Third, the survey questionnaire was designed by the authors of this study; therefore, validation of the survey measure was not confirmed. However, there are only a few validated questionnaires for occupational satisfaction [37] and we were not aware of questionnaires that best-matched the aim of the present study. Fourth, this study was a self-report design. It is difficult to evaluate the actual level of symptoms or presence of factors with high accuracy. Fifth, the R^2^ values were not high in the multiple regression analyses that we performed in the present study. This is a possibility that several items would be confounding factors, such as number of patients who committed suicide.

## Conclusions

The present study found that nearly one-third of psychiatrists who participated screened positive for depression. Higher occupational satisfaction was associated with a less severe depressive state under moderate levels of occupational stress. Enhancing occupational satisfaction by discussing individuals’ interest in work with a supervisor as well as obtaining opportunities for career development may help to prevent the development of depressive symptoms among healthcare professionals. Additional prospective studies are needed to confirm the present findings.

## Additional file

Additional file 1:
**Survey Questionnaire.**
