# Supplementary material for: Mental health of Japanese psychiatrists: the relationship among level of occupational stress, satisfaction and depressive symptoms
Source: BMC Res Notes. 2015 Mar 26;8:96. doi: 10.1186/s13104-015-1054-7 (PMC4409783; doi:10.1186/s13104-015-1054-7)
Supplement: Additional file 1: — Survey Questionnaire. [file 13104_2015_1054_MOESM1_ESM.docx]

**Additional file 1:** Survey Questionnaire

1. **Please place a mark on the scale below to indicate your current level of satisfaction at your work.**

(the best possible is marked 100 and the worst possible is marked 0)


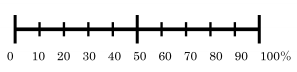


1. **Please place a mark on the scale below to indicate your current level of stress at your work.**

(the best possible is marked 100 and the worst possible is marked 0)


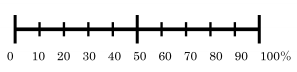


1. **Please tick the option that best describes your current workplace health.**

-Agree / somewhat agree / somewhat disagree / disagree-

1. You have to do an enormous amount of work. (*work overload*)
2. You cannot complete all your work in the allotted time. (*insufficient time*)
3. You have to focus your attention quite a lot. (*necessity to concentrate one's attention on work*)
4. You can work at your own pace. (*ability to work at one’s discretion*)
5. You can decide the order in which you do your work and the way you do it.(*ability to make one’s decisions*)
6. You can reflect your own opinions on the workplace’s work strategy. (*ability to reflect one’s opinion*)
7. You can often communicate with supervisor. (*ease of communication with supervisor*)
8. You can strongly rely on supervisor if you have some trouble.(*ability to rely on supervisor*)
9. Your supervisor kindly spend his/her time on your private problem.(supervisor spends time on one’s personal issues)
10. You can often communicate with co-worker. (*ease of communication with colleague*)
11. You can strongly rely on co-worker if you have some trouble. (*ability to rely on colleague*)
12. Your co-worker kindly spend his/her time on your private problem.(*colleague spends time on one’s personal issues*)
13. **Please tick the option that best describes your current workplace health.**

- Never / Seldom / Sometimes / Often / Always -

1. You often feel insecure during patient care. (*workplace insecurity*)
2. You often see patients that you have great interest. (*interest towards work content*)
3. Category of your annual salary.(*monetary reward*)

less than \5M / \5M-\10M / \10M-\15M / \15M-\20M / more than \20M

1. You often satisfy with your workplace’s clinical meetings and rounds. (*opportunity for growth and career development*)
2. You often receive satisfactory evaluation from patients (*gratitude and respect from patients*)
